# Preadolescent Children Using Real-Time Heart Rate During Moderate to Vigorous Physical Activity: A Feasibility Study

**DOI:** 10.2196/58715

**Published:** 2025-03-06

**Authors:** Lincoln Lu, Danielle E Jake-Schoffman, Hannah A Lavoie, Maedeh Agharazidermani, Kristy Elizabeth Boyer

**Affiliations:** 1 LearnDialogue Lab Computer and Information Science and Engineering University of Florida Gainesville, FL United States; 2 Exhale Lab Health and Human Performance University of Florida Gainesville, FL United States

**Keywords:** smartphone app, physical activity, heart rate, wearable sensors, youth, commercial wearable device, Garmin, mobile phone

## Abstract

**Background:**

Given the global burden of insufficient physical activity (PA) in children, effective behavioral interventions are needed to increase PA levels. Novel technologies can help expand the reach and accessibility of these programs. Despite the potential to use heart rate (HR) to target moderate- to vigorous-intensity PA (MVPA), most HR research to date has focused on the accuracy of HR devices or used HR for PA surveillance rather than as an intervention tool. Furthermore, most commercial HR sensors are designed for adults, and their suitability for children is unknown. Further research about the feasibility and usability of commercial HR devices is required to understand how children may use HR during PA.

**Objective:**

This study aimed to explore the use of a chest-worn HR sensor paired with a real-time HR display as an intervention tool among preadolescent children and the usability of a custom-designed app (Connexx) for viewing real-time HR.

**Methods:**

We developed Connexx, an HR information display app with an HR analytics portal to view HR tracking. Children were recruited via flyers distributed at local public schools, word of mouth, and social media posts. Eligible participants were children aged 9 to 12 years who did not have any medical contraindications to MVPA. Participants took part in a single in-person study session where they monitored their own HR using a commercial HR sensor, learned about HR, and engaged in a series of PAs while using the Connexx app to view their real-time HR. We took field note observations about participant interactions with the HR devices. Participants engaged in a semistructured interview about their experience using Connexx and HR during PA and completed the System Usability Scale (SUS) about the Connexx app. Study sessions were audio and video recorded and transcribed verbatim.

**Results:**

A total of 11 participants (n=6, 55% male; n=9, 82%, non-Hispanic White) with an average age of 10.4 (SD 1.0) years were recruited for the study. Data from observations, interviews, and SUS indicated that preadolescent children can use real-time HR information during MVPA. Observational and interview data indicated that the participants were able to understand their HR after a basic lesson and demonstrated the ability to make use of their HR information during PA. Interview and SUS responses demonstrated that the Connexx app was highly usable, despite some accessibility challenges (eg, small display font). Feedback about usability issues has been incorporated into a redesign of the Connexx app, including larger, color-coded fonts for HR information.

**Conclusions:**

The results of this study indicate that preadolescent children understood their HR data and were able to use it in real time during PA. The findings suggest that future interventions targeting MVPA in this population should test strategies to use HR and HR monitoring as direct program targets.

## Introduction

### Background

Physical activity (PA) levels are insufficient across most of the world’s population [1], constituting a persistent problem in global health. PA engagement in the United States is reflective of global trends: only 25% of US children meet the minimum daily recommended amount of moderate- to vigorous- PA (MVPA) [2]. PA behaviors and insufficient PA developed during childhood often worsen during adolescence if not addressed through intervention [3]. Insufficient PA is also a risk factor for obesity, a condition related to various comorbidities [4] that can persist into adulthood [5,6]. Alarmingly, children who develop obesity at a young age are more likely to have obesity into adulthood [7]. Therefore, addressing obesogenic factors (eg, PA) during childhood has the potential to positively impact long-term overall health [8,9].

Effective behavioral interventions are essential to address insufficient PA, particularly before the significant decline that often occurs during adolescence, around the age of 13 years [10]. Novel technologies can help expand the reach and accessibility of PA promotion efforts. To date, most research leveraging commercial PA devices has focused on adult populations [11], although some studies focusing on children have examined step counts as a PA outcome [12]. Step count measures basic movement, but this metric insufficiently captures the intensity of movement and, therefore, may be inadequate for measuring or promoting MVPA.

In contrast to step count, heart rate (HR) holds great promise for measuring MVPA. Moreover, it is possible that novel technologies allowing children to view their HR in real time could foster PA awareness and engagement. However, this great potential is understudied; a recent systematic review found only two studies that examined the use of existing commercial sports-style HR sensors for use in preadolescent children [13]. The studies identified by the systematic review focused on whether children would wear an HR sensor on their chest paired with a wrist-worn HR display over a long time period [14] and the accuracy of commercial HR sensors on a preadolescent child’s wrist against a medical-grade control [15]. Another recent study involved the use of chest-worn HR sensors paired with a wrist-worn HR display to monitor PA in adolescents and young adults. However, the study did not provide participants with education on HR or guidance on effectively using HR to achieve MVPA goals [16].

These studies demonstrate the accuracy of commercial HR sensors and displays for preadolescent children. The adoption of devices capable of displaying real-time HR information is growing in this age group, matching trends across the demographic spectrum. Recent statistics indicate wide adoption of smartphones: 97% of Americans own a smartphone [17], and ownership is equitable across racial and socioeconomic groups [18]. Statistics from a survey conducted in July 2019 reported that approximately 21% of the US population owned a smartwatch [19]. Smart device adoption is growing among preadolescent children in the United States; by their teenage years, 95% of children either have their own smartphone or have access to one through their family [20]. Despite the growing ubiquity of these devices, research has only focused on the technical ability of smart devices to surveille user HR [16]. Research should now be conducted to explore the use of real-time HR as an intervention tool to target specific MVPA goals.

### Objectives

Pairing chest-worn HR sensors with existing smart devices in the form of smartphones and smartwatches is a novel opportunity that has not been sufficiently explored. However, providing real-time HR information without exploring preadolescent children’s ability to understand and use HR in real time to effectively target MVPA would only replicate existing studies with new hardware [16]. Therefore, we provided participants with information about HR and how to target different HR zones during PA. This study primarily aimed to explore whether preadolescent children can use real-time HR information to target specific MVPA goals. A chest-worn HR sensor was paired to a smart device (eg, smartphone or smartwatch) that displayed real-time HR information. As the HR sensors used in this study were originally designed for adult use, a secondary objective of this study was to explore whether preadolescent children could feasibly use a chest-worn HR sensor paired with a smart device.

Another secondary objective of this study was to explore the usability of a custom app (Connexx) developed by the research team. As part of a broader program of research, the team is exploring the impact of collaboration on PA, and one facet of this research explores collaborative pairs in a family dyad consisting of a parent or guardian and child. Dyads collaborate by viewing each other’s real-time HR information during PA. There are currently no commercial apps capable of transmitting and displaying real-time HR information between two users. We developed the Connexx app with this functionality. However, a key aim of this study was to explore the usability of Connexx for preadolescent children, who represent half of the intended pair in parent or guardian and child collaborative dyads. Thus, this study explored the usability of the Connexx app in solo mode to establish its feasibility in use with preadolescent children.

## Methods

### Study Setting

This feasibility study was conducted at the University of Florida, Gainesville, Florida. Data collection occurred between October 2023 and February 2024.

### Ethical Considerations

The study was approved by the University of Florida Institutional Review Board (IRB202301463) and complies with the ethical guidelines mandated therein. Informed consent was obtained from a parent or guardian electronically, and written assent was obtained from the participants themselves at the start of each study session. Audio and video recordings from study sessions were stored on secured servers mandated as part of the University of Florida Institutional Review Board parameters. A US $25 Amazon e-gift card was provided to the participant’s parent or guardian at the conclusion of the study session.

### Study Equipment

**Figure 1:**
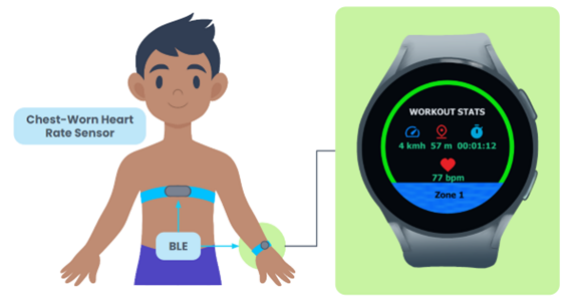
Smartwatch with solo workout screen connected to Bluetooth heart rate sensor. bpm: beats per minute; BLE: Bluetooth Low Energy.

Several pieces of commercial technology were used to measure and display HR information (Figure 1). HR was measured using a sport-style chest-worn HR sensor (Garmin HRM-Pro Plus), and HR information was transmitted via Bluetooth Low Energy and displayed on either a smartphone or smartwatch. The smartphone used was a Google Pixel 4A, selected due to its low cost and the ability of Android phones to sideload apps, allowing us to install the Connexx app on the smartphone. The smartwatch used was a Samsung Galaxy Watch 4, selected for its Android-based operating system, which allows the Connexx app to be modified from the smartphone version to be compatible with the Galaxy Watch 4.

**Figure 2:**
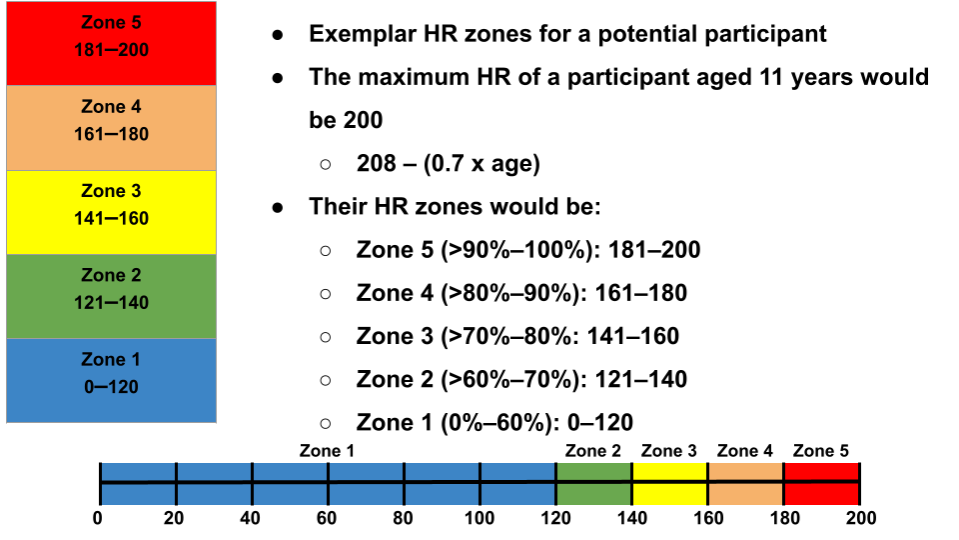
Exemplar heart rate (HR) zone explanation card for a participant aged 11 years.

This study used HR rather than accelerometry readings to measure MVPA. HR was chosen because it can be used to display effort in real time across various types of activities, which can contribute to a more accurate measure of the amount of time spent in MVPA [16]. We developed the Connexx app to display real-time HR information between two users. The Connexx app transmits real-time HR information between two devices via a traditional Bluetooth connection. However, Connexx is also capable of operating in solo mode, displaying the real-time HR of a single user, as we did in this study (Figure 1). Information on the user’s current speed, distance covered during the workout, and workout duration was displayed along the top row of the Connexx solo workout screen. Speed and distance fields can be toggled between metric and imperial units by tapping on the display. The middle row of the workout screen displays the user’s real-time HR. Connexx is able to display real-time HR information as either beats per minute (BPM) or as a percentage of maximum HR; users can toggle between these two display options by tapping on the HR display. A color-coded HR zone indicator is displayed below the HR field. This color coding matched the colors on HR exemplar cards used to explain HR information to participants (Figure 2). In this study, maximum HR was calculated using an established formula that is accurate for preadolescent children: 208 – (0.7 × age) [21]. The Connexx app automatically calculates HR zones using a straightforward 5-zone division based on the percentage of maximum HR, selected because there was no clear consensus or definitive definition of specific HR zones for preadolescent children [22-24] (zone 1: 0%-60%, zone 2: >60%-70%, zone 3: >70%-80%, zone 4: >80%-90%, and zone 5: >90%-100%). Furthermore, the Connexx app has an analytics portal; after the app records a workout, the information is sent to this portal, capable of displaying data from the workout on a graph.

### Participants, Eligibility, Recruitment, and Screening

This study aimed to enroll a sample of 12 diverse preadolescent children to complete the full protocol. This sample size was chosen based on best practices in usability testing, showing that a sample of 10 to 12 participants is able to identify most major user issues [25]. Children were eligible to participate if they were aged 9 to 12 years and in good health with no physical conditions, injuries, or other conditions precluding them from engaging in MVPA for up to 30 minutes (assessed via a modified Physical Activity Readiness questionnaire for everyone [26]). We sought an equal number of male and female participants and equal distribution between Black, Hispanic, and non-Hispanic White participants (the three largest racial or ethnic groups in the local area).

Recruitment targeted parents of potentially eligible children using an electronic flyer distributed via local public elementary and middle schools, social media posts, and word of mouth. The flyer provided basic information regarding the study, eligibility criteria, compensation (US $25 Amazon e-gift card), and a link or QR code to a Qualtrics screening questionnaire. Parents or guardians completed the eligibility questionnaire, which included demographic information about their child. If the child was eligible, the parent or guardian was contacted via email to sign an electronic consent form through REDCap (Research Electronic Data Capture; Vanderbilt University). Once consent was obtained, a study team member (LL) scheduled the child for a study session.

### Study Session

**Figure 3:**
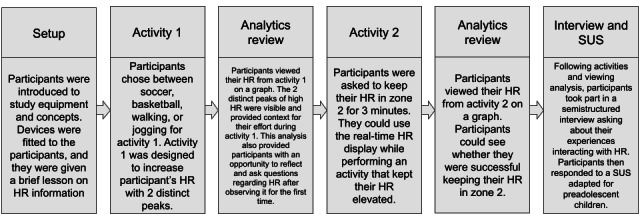
Study session activities flowchart. HR: heart rate; SUS: System Usability Scale.

Study sessions were conducted at an indoor multiuse facility at the University of Florida. The space measures approximately 1021 square meters (27.7 meters by 36.9 meters) and can be used for a variety of uses, including basketball and volleyball; it has a maximum capacity of 1140 people. The study session began with obtaining assent for participation from the preadolescent child and followed the format outlined in Figure 3.

### Device Setup

Following assent, participants were fitted with Rode wireless lapel microphones. A researcher (LL) explained the study equipment and demonstrated the functionality of the chest-worn HR sensor. Parents aided participants in putting on the HR sensor. The session was recorded with a video camera. Next, participants chose between a smartphone and a smartwatch to view their real-time HR information. The Connexx app was available to all participants on either a smartphone or a smartwatch. However, all participants chose to use the smartwatch. The participants were guided through the process of entering their study information, such as their research identification number, age, and maximum HR, into the Connexx app. A workout was then initiated on the app, allowing participants to view their HR in real time, with more HR information provided to them.

### HR Information

HR and HR zones were explained to the participants using a real-time HR display in Connexx (Figure 1) and an exemplar HR information card (Figure 2) based on their age (Table 1). Additional language used to explain HR and HR zones is provided in Multimedia Appendix 1. The goal of the brief explanation was to frame the upcoming activities in a way that participants could understand in relation to their HR zones. The researcher encouraged the participants to ask questions during the HR explanation.

**Table 1:** Example of the 5-zone heart rate (HR) categorization and description for participants aged 11 years.

HR zones	BPM^a^	Zone effort category	Example activities associated with the zone	Description of the zone
Zone 5	181 to 200	Maximum	Very high effort activities, which are often repeated (eg, sprinting in games such as tag multiple times in a row)	Extremely difficult to hold a conversation and most people must wait until after this effort to speak
Zone 4	161 to 180	Vigorous	Vigorous activities, such as playing a sport (eg, soccer or basketball), strenuous hiking, running, or climbing flights of stairs continually at a brisk pace	Difficult to hold conversation and often can only squeeze in short 1- or 2-word responses around breathing
Zone 3	141 to 160	Endurance	Shorter vigorous activities or long endurance activities (eg, climbing 2 to 3 flights of stairs or walking or hiking for an hour)	Slight difficulty to hold a normal conversation and speech is negotiated around breathing
Zone 2	121 to 140	Low endurance	Watching an exciting program and slow walk for short distances (eg, walking to the kitchen and walking around the block at a slow pace)	Can hold normal conversations without thinking about breathing
Zone 1	≤120	Rest	Sleeping, sitting and reading a book, or watching television	Can hold normal conversations without thinking about breathing

^a^BPM: beats per minute.

### Activities

Next, we outlined the structure of the study to participants (Figure 3). The study consisted of 2 activity intervals; activity 1 lasted for 10 minutes and activity 2 lasted for 3 minutes, each followed by a brief rest period during which participants could view their HR information via an analytics portal on a laptop.

#### Activity 1

This study primarily aimed to explore whether preadolescent children can use real-time HR information to target specific MVPA goals. Activity 1 was designed to provide participants with an opportunity to observe their HR as it elevated through several different zones aligning with MVPA, providing practical, experiential use of HR information for participants. For activity 1, participants chose from playing basketball or soccer, or walking or jogging around the gym space. A researcher moderated participant effort during activity 1 by speeding up or slowing down the activity to take participants through a range of HR values. During this activity, the researcher asked participants to report their real-time HR at regular intervals. Querying for HR regularly was intended to help participants notice how their HR responded to various activity levels. Activity 1 was designed to increase participants’ HR through multiple HR zones twice. Participants viewed their HR through the Connexx app while engaged in PA. After completing activity 1, the researcher guided participants through ending the workout on the Connexx app, and then, participants viewed a graphical representation of their HR in a Connexx analytics portal. We guided participants through the HR observation process, drawing attention to how HR can vary at rest, how HR responds to effort levels during PA, and how the body feels at various HRs.

#### Analytics Reflection

**Figure 4:**
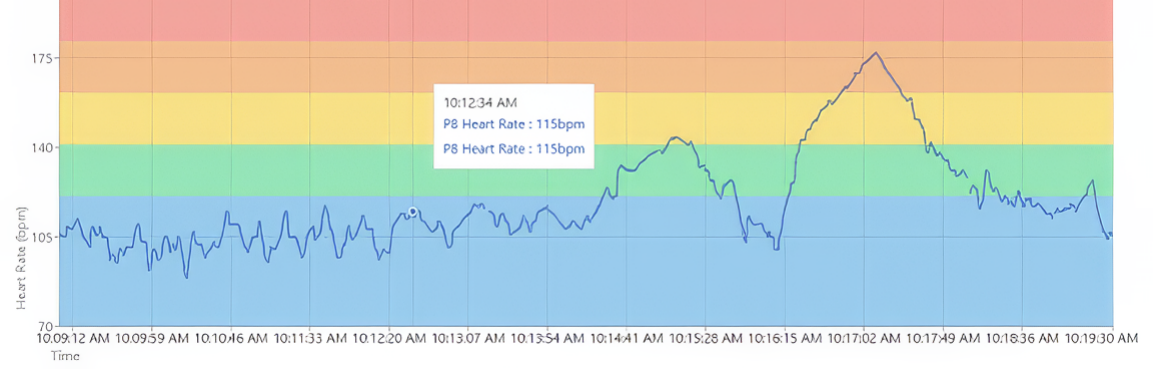
Sample heart rate graph following activity 1. bpm: beats per minute.

**Figure 5:**
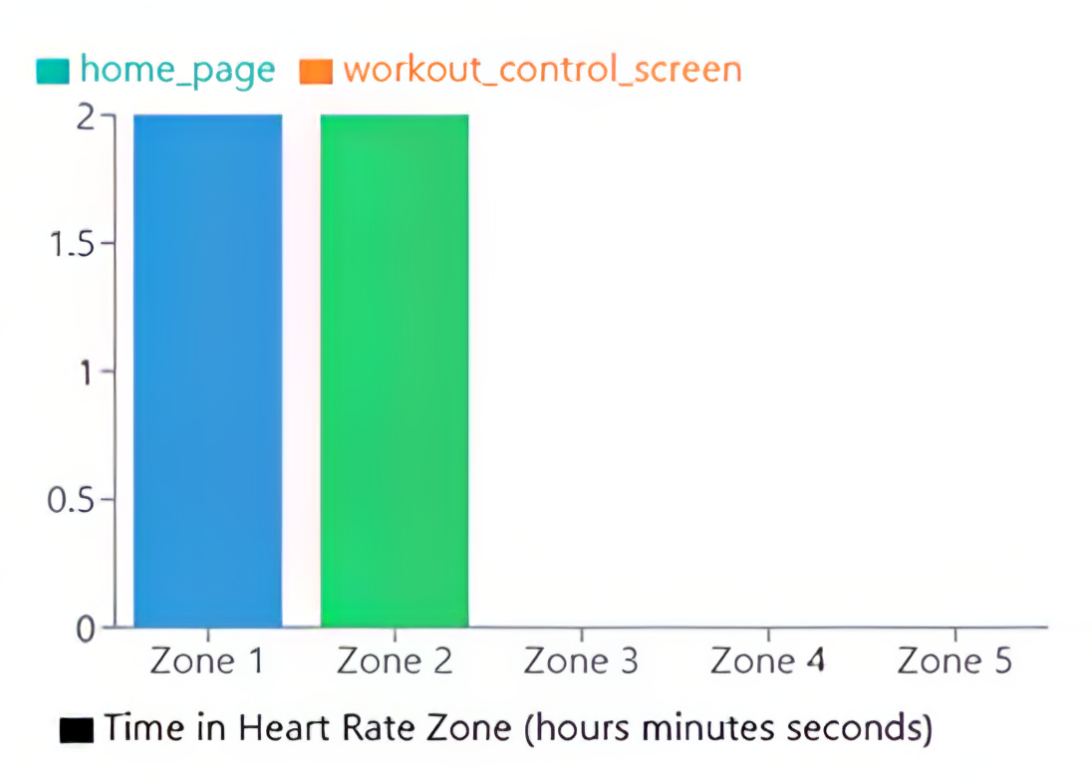
Sample heart rate zone cumulative time.

Once a workout ended on the Connexx app, workout data were uploaded to the analytics portal. The analytics portal displayed the participant’s color-coded HR zones in the background of the graph, with HR in BPM on the vertical axis and duration on the horizontal axis (Figure 4). The researcher pointed to the connection between the HR graph and PA level: the more intense the PA level, the steeper the HR graph line became. Furthermore, the researcher pointed to how variable HR was: when at rest, HR did not stay at a consistent BPM; rather, it varied up and down slightly. In addition to the HR timeline graph, the analytics portal also presents an HR zone breakdown, which is a bar graph showing the total cumulative time the participants’ HR spent in each zone (Figure 5). Participants were encouraged to ask questions throughout the process of viewing their HR graph.

#### Activity 2

Activity 2 was designed to determine whether participants could make use of the tools provided to them so far (such as the information they have learned about HR, the HR sensor they were wearing, and the Connexx display, which provided a real-time display of their HR in BPM and HR zones) to maintain their HR in a targeted zone. Participants were asked to maintain their HR in zone 2 while engaged in the PA of their choice (basketball, soccer, walking, or jogging). Zone 2 was chosen because it was accessible to all participants, regardless of their fitness level, and they had to use the tools provided to maintain zone 2 HR, as they could easily exceed zone 2 HR by exercising too intensely. Participants could use the Connexx display to monitor their own HR and HR zone in real time to maintain zone 2 HR. The only prompts we provided were for timing (eg, when each minute elapsed). After 3 minutes, the participant was invited to save and end their workout on the Connexx app and view their HR graph for the second interval.

#### Analytics Review

**Figure 6:**
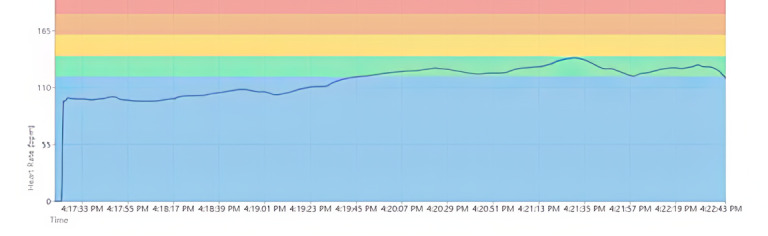
Sample heart rate graphical displays following activity 2. bpm: beats per minute.

The analytics portal displayed whether participants were successful in maintaining their HR in zone 2 (Figure 6). The HR zone breakdown also indicated the total time participants spent in each HR zone.

### Interview and System Usability Scale

After completing the 2 activities, participants engaged in a semistructured interview about their experiences. The interview explored participants’ preference for smartwatch or smartphone, the comfort of commercial chest-worn HR sensors and smart devices, the participant’s real-time actions to adjust their HR to meet a target goal, what HR zones mean to them, their preference for HR zones, whether they had previous experience with HR, and their enjoyment and potential future use of the Connexx app (refer to Multimedia Appendix 2 for the full interview script). During the interview, relevant objects and visual aids were presented to the participants as needed (eg, smartwatches and HR zone cards).

Finally, after the interview, participants filled out a modified version of the System Usability Scale (SUS) specifically modified and validated for use with preadolescent children [27]. This 10-item scale is used to calculate a usability score between 0 and 100. The commonly accepted threshold for a usable technology is a score of 68 [28].

### Observational Data Collection Measures

We conducted observational data collection during each study session. In the event of recording capture device failure, a physical copy of the start and end times of each activity and a log of the participant’s HR report and its corresponding value during the study session provide an overview of the participant’s HR. Fortunately, this backup was not necessary during analysis.

The researcher who primarily interacted with the participants recorded field notes after each session. Notes focused on participant interaction with the instruments that might not be captured through the interview or SUS questionnaire, such as whether additional adjustments to the chest-worn HR sensor were required and how quickly participants adapted to the input methods for the Connexx app.

### Analysis

Demographic data were summarized with descriptive statistics. Observational data were collated with the session transcripts to provide more depth and nuance to the thematic analysis. Additional observations that were not captured in the transcripts or SUS results are included as separate findings in the Observational Findings section.

Interviews were recorded and automatically transcribed via Microsoft OneDrive; we reviewed and revised autogenerated transcripts. Three researchers (LL, MA, and HAL) with qualitative research experience performed an inductive thematic analysis [29]. They followed the 6 steps of thematic analysis: data familiarization, initial code generation, searching for themes, theme review, defining and naming themes, and reporting [30,31]. While this study was designed with several objectives, we remained open to accepting any informative findings in the data. LL, MA, and HAL collaborated to familiarize themselves with the data and generate codes using a sample of the first 2 participant transcripts. The researchers worked together to search and identify themes, creating a codebook. The team then coded transcripts independently, meeting after completing the third and fourth participant transcripts to refine the codebook. Then, they coded the rest of the participant transcripts independently. Intercoder reliability was calculated using Krippendorff α [32] on a sample of 4 independently coded transcripts across 3 coders using the SPSS software (IBM Corp) extension developed by Hayes and Krippendorff [33]. Krippendorff α reliability estimate for the 3 coders was 0.907 (95% CI 0.8348-0.9621), corresponding to a high degree of agreement among them.

## Results

### Participant Demographics

A total of 11 preadolescent children with an average age of 10.5 (SD 1.0) years participated in this study, of whom 6 (55%) were male and 9 (82%) were non-Hispanic White. While the team sought to recruit a sample with equal numbers of male and female children and an even distribution of races and ethnicities, this diversity was not achieved in the final sample.

### Observational Findings

Several observations demonstrated that preadolescent children can use real-time HR information to target specific MVPA goals. Participants shared a strong interest in their real-time HR information; some participants were quick to explore how their HR would respond to changes in PA. Participants looked at their HR information multiple times after initially starting a workout in the Connexx app and observed how controlling their breathing or fidgeting in their chairs may impact their HR. Their ability to use real-time HR information was further demonstrated by the actions of participants in activity 2. For activity 2, participants were asked to maintain their HR in zone 2 for 3 minutes of PA. A total of 7 participants successfully used the Connexx display to hold their HR in zone 2. Of the remaining 4 participants, 1 (25%) initially ran at a high intensity that raised her HR above zone 2; however, throughout the rest of the 3-minute activity, she ran progressively slower to bring her HR back into zone 2. The 3 (75%) other participants took the opportunity to exert themselves as hard as possible in an attempt to achieve the highest HR reading instead of holding their HR in zone 2. These participants knew how to use the Connexx display to observe their HR in real time, as they looked at their smartwatch throughout activity 2. However, these 3 participants had a strong desire to see how high their HR could go. The parent of 1 of these 3 participants noted that she was highly competitive and always wanted to outcompete her older brother (who was also a participant in this study). One of the participants who successfully held his HR in zone 2 during the second activity demonstrated a similar desire to achieve a high HR value when he asked to be allowed to run around the gym space after completing the study so that he could see how fast his HR could read in the Connexx app.

Regarding hardware suitability for the children, all 11 participants chose to use a smartwatch to observe their HR in real time, and the watch straps successfully fit across a range of wrist sizes. However, there were some challenges with the chest-strap HR sensor. Two (18%) of the 11 participants needed the strap on the HR sensor to be shortened beyond its normal range to fit securely around their bodies. In both cases, the strap was shortened by <5 cm (by folding the excess fabric over and securing it with a safety pin) from the lower limit of the original strap range.

Furthermore, participants were able to quickly learn how to use the Connexx app. All participants were able to report their real-time HR and HR zone when prompted. Several participants (7/11, 64%) were also able to navigate the Connexx app themselves after the initial setup. They could swipe through the different screens in the app and start and end workouts themselves, and 2 (18%) out of 11 participants were able to navigate the text input interface with minimal guidance from the researcher. However, participants identified a substantial issue with the Connexx app in its current state. During the activities, participants noted that the font size of their HR display was too small. This was of particular concern during activity 1, as participants were moving vigorously at points to raise their HR through multiple HR zones. Participants had to adopt several different strategies to be able to see their HR values, including bringing the watch close to their face or slowing down their movement. The small font size of the Connexx app contributed to interruptions in the MVPA participants were engaged in.

### Interview Findings

The interview provided rich data that generated themes related to using commercial devices to observe real-time HR to target specific MVPA goals. Three major themes emerged: one focused on real-time use of HR, one related to HR knowledge, and another on device and app use. Specific subthemes ranged from reports of real-time HR to preferences for the construction of HR zones. Themes, subthemes, definitions, and sample quotes are provided in Table 2. Major subthemes are described in the following sections in detail.

**Table 2:** Interview themes and sample quotes.

Theme and subtheme		Subtheme definition	Sample quotes
**Real-time sensemaking of HR^a^**			
	Reporting HR and HR zones	Declarations of participant’s real-time HR and HR zone	“Oh, I’m at Zone 4!... (which is) 160 (BPM).” [Participant 2]
	Real-time HR use	How participants make use of real-time HR during activities	“I just looked at the watch a couple of times throughout the exercise and I saw Zone 2 and I’m like, OK, I’m, I was and I, you know, thought that I was doing good. So, I just kept it at that pace.” [Participant 9]
	HR and correlation to body sensations	How participant’s body feels at different HR and HR zones	“I feel like I have to breathe more, but it mostly feels similar.” [Participant 1]
**HR-related knowledge**			
	Confusion regarding HR	Confusion about HR or HR zones	“I was just walking up and going over there to play soccer and it (HR) was (already) at 117.” [Participant 4]
	HR as an achievement	Desire for a high HR or HR zone displayed	“... with five, it’s, I just like feel kind of accomplished.” [Participant 8]
	Previous knowledge of HR	Previous experience learning about HR	“In elementary school in PE class... count... how many beats we have in six seconds? And then he would tell us to, like, multiply it by 10 and that’s beats per minute” [Participant 4]
	HR zone preferences	Preference for HR zones, 3 larger or 5 smaller zones	“I personally like breaking stuff down to like smaller pieces and understanding it better.” [Participant 9]
**Device and app**			
	Device user experience	Comments on function and design of the device	“The smartwatch, you could just turn it over to look...” [Participant 7]
	Device comfort or discomfort	How the devices felt for participants	“Wasn’t too tight, too heavy... it feels comfortable.” [Participant 3]
	App usability	Like or dislike the app and willingness to use (or not use) the app in the future	“Probably just for like for fun, just to see (my HR).” [Participant 7]

^a^HR: heart rate.

### Using Real-Time HR Information

During the interview, participants were asked about their strategies for adjusting their efforts to meet specific MVPA goals, including holding their HR in zone 2 for 3 minutes in activity 2. Participants who successfully held their HR in zone 2 discussed how they adjusted their effort based on the HR display (coded as “Real-time HR use” in Table 2):

I tried to keep the same speed when it was in Zone 2, because if I got faster, it [referring to his HR] will go higher, and if I went slower, it will go to Zone 1.

Participant 3

Often, this involved reducing their effort to not exceed the upper limit of zone 2 (coded as “HR and correlation to body sensations” in Table 2):

I tried using less power for [basketball] shots and like, try and walk or running across slower to conserve [energy].

Participant 1

The participants who were successful in keeping their HR steady in zone 2 used their smartwatch to adjust their effort much more often:

I just kept looking at the watch.

Participant 2

Some participants (2/11, 18%) noticed that their HR would change even when they did not think their actions should correspond to the change (coded as “Confusion regarding HR” in Table 2):

I was just walking up and going over there to play soccer and it [HR] was [already] at 117.

Participant 4

However, noting this unexpected result demonstrated that the participant was actively making connections between their PA and the displayed HR.

Not all participants were able to successfully keep their HR in zone 2 during activity 2. These participants acknowledged that they knew the goal was to maintain their HR in zone 2, but they wanted to achieve the highest HR possible. When asked what their HR was, participant 1 stated that “it just hit Zone 4, it is 161... I can go way faster than that!” (coded as “HR as achievement” in Table 2), and participant 8 expressed interest in his HR throughout the course of the study session. After finishing activity 1, he told the researcher that he “would like to see what it (his HR) looks like on a graph.” Furthermore, after activity 2, where he was not successful in keeping his HR in zone 2, he told the researcher that he did “want to see how long I was in [zone] 5 for.”

### HR-Related Knowledge

In addition to findings on the specific research objectives, several themes emerged during the inductive thematic analysis process.

#### Previous Experience With HR

Four (36%) of the 11 participants had learned about HR in school before taking part in the study (coded as “Prior knowledge of HR” in Table 2); however, none of the participants had a complete understanding of HR. Learning about HR in school was summed up by a participant as follows:

[We would] do jumping jacks or jog in place for like 30 seconds, 20 seconds, and then... count our heart, how many beats we have in six seconds, and then he would tell us to, like multiply it by 10, and that’s beats per minute.

Participant 4

None of the participants had used a chest-worn HR sensor before the study.

#### HR Zones

Participants were asked to describe what HR zones meant in their own words, and overall, they were able to provide a basic description of each zone. A participant explained the zones in a manner similar to how we had explained them before activity 1, as follows:

Zone 1, just calm, sitting down and not doing anything. Zone 2 I might be walking around, and I don’t know. Zone 3 I would start to feel really hard to breathe and a little faster [breathing]. Zone 4, I might be breathing kind of heavier and having to slow down a little bit.

Participant 4

Other participants described the zones in relation to their own experiences during the study:

[Zone] 4 was like, I got to 4 when I was like shooting the ball and running.

Participant 7

Participants were asked whether they had a preference for the 5 HR zones used in this study or a more simplified 3 HR zones (coded as “HR zone preferences” in Table 2). Three (27%) of the 11 participants preferred the simplicity of having fewer zones:

It would be easier to look at if there was just easy, medium, hard. It would be easier to keep it in the easy-medium zone [referring to Zone 2].

Participant 4

The other participants (8/11, 73%) preferred 5 HR zones. Reasons for preferring 5 zones differed between participants; some preferred the greater granularity offered by more zones, and others preferred more HR zones as it provided a sense of achievement. A participant explained the meaning of having more HR zones as follows:

It would narrow it down and be easier just to keep it at that zone.

Participant 6

Another participant explained his preference for more HR zones as follows:

Oh, it’s just like an achievement. It kind of feels like an achievement.

Participant 7

I just want to know if I’m in Zone 5, because, like, it’s exciting.

Participant 7

### Feasibility of Chest-Worn HR Sensors Paired to Smart Devices

#### Device Preference

To explore device preferences, participants were asked why they chose to use a smartwatch over a smartphone to observe their HR during PA (which all participants did). While only 2 (18%) of the 11 participants had consistently used a smartwatch before the study (one had their own, and another used their father’s), all participants commented on the greater convenience they could foresee with a smartwatch, particularly while engaging in PA (coded as “Device user experience” in Table 2):

The smartwatch, you could just turn it over to look. But for a smartphone, you have to grab it, then you have to [mimes the action of pulling a phone out of their pocket to look at it]. If you’re like working out, and you want to know [your HR] you could just look.

Participant 7

#### Device Comfort

When asked about the comfort or discomfort of the devices, all participants stated that the smartwatch felt comfortable (coded as “Device comfort or discomfort” in Table 2):

I’m used to wearing watches, so it’s pretty normal for me to put one [smartwatch] on.

Participant 1

While there was some initial discomfort with the chest-worn HR sensor, it soon became unnoticeable:

When I first put it on, it kind of feels weird. But I got used to it when I used it for quite a while.

Participant 3

Only one participant reported continued discomfort and a desire to take off the HR sensor before the conclusion of the interview:

It was hot and kind of squeezing, so you get uncomfortable feeling.

Participant 7

Furthermore, one participant noted that using a softer fabric for the strap would increase the comfort of the instrument:

If like the material is different, so you didn’t feel it as much. Like if it’s like a smoother material or something maybe not as thick.

Participant 6

### Usability of the Connexx App

Overall, participants were enthusiastic about the Connexx app and the ability to observe their own HR data (coded as “App usability” in Table 2). Participant 7 represented most participants when he declared that using Connexx “was kind of fun!” However, the small font size on the Connexx display made it challenging to view real-time HR while engaged in PA. For instance, participant 1 stated that what he “found frustrating was when we were running that when I wanted to check my HR, I couldn’t see it straight because of it shaking.” Many participants (9/11, 82%) expressed interest in using the Connexx app in the future. The final response of participant 11 to a series of questions regarding the Connexx app and the ability to view her own HR was that she could “use it for playing games” with her friends. Similarly, participant 7 noted that he would like to use the Connexx app again in the future, “probably just for like for fun.”

### SUS Findings

The Connexx app can be considered highly usable in its current iteration; participants rated the Connexx app with an average score of 77.1 (SD 13.7), well above the threshold score of 68 [28]. However, feedback from this study will contribute to its future refinement to further increase usability.

## Discussion

### Principal Findings

While significant research has demonstrated the accuracy of commercially available HR devices for children [15], there has been insufficient research on how real-time HR information can be used to target MVPA goals. In this study, preadolescent children were provided with a basic lesson about their HR and HR zones, and then, they engaged in 2 PA sessions. Participants demonstrated an understanding of the HR display during PA by effectively using it to regulate their efforts. Therefore, real-time HR information can be used by children as a tool to meet PA guidelines more effectively; children can monitor their own HR information to ensure that they are engaged in MVPA. In addition, this study provides insight into the feasibility of HR sensors and smart devices, smart device preferences, the usability of a custom HR app, preadolescent children’s previous HR experiences, and HR zone preferences.

### Preadolescent Children Using Real-Time HR Information to Target Specific MVPA Goals

The findings of this study are unique in research on HR device use. Previous research often focused exclusively on accuracy [34], overlooked how users interact with commercial fitness trackers [35], or did not include HR information [36] in the design. In fact, preadolescent children in one study complained that the only real-time information they could see were step count and distance [36]. Participants in a study that paired chest-worn HR sensors with a wrist-worn display regulated their effort based on running speed; these participants did not use their available real-time HR information to adjust their effort [16]. Participants in this study demonstrated their ability to use real-time HR and HR zone information during PA by successfully keeping their HR in zone 2. We observed participants adjusting their effort while exercising (during activity 2) to keep their HR in zone 2. Participants confirmed in the interview that they adjusted their movement speed based on their real-time HR display, demonstrating an ability to use their real-time HR information to target specific MVPA goals during the study session.

### Feasibility of Chest-Worn HR Sensors Paired to Smart Devices

Results of this study suggest that chest-worn HR sensors are feasible for use by preadolescent children. While some participants (2/11, 18%) mentioned slight discomfort when first putting on the chest-worn HR sensor, this did not seem to be an ongoing issue and was not brought up during activity 1 or 2. This study provides a more comprehensive investigation into the comfort and usability of these devices compared to previous research. One study that examined the use of a chest-strap HR sensor in conjunction with a wrist-worn device for preadolescent children focused on the accuracy of measurement rather than how preadolescent children felt about using these devices [14]. Another study focused on the accuracy of commercial devices for HR measurements, in this case, wrist-worn devices, without consideration for the comfort of the device [15]. Participants in this study were undergoing surgery, which may not be an appropriate situation to consider the comfort of an HR monitoring device. Furthermore, previous research that focused on the comfort of devices [16] examined an older population, whose more developed bodies may be better suited for devices typically designed for adults. However, the findings of this study indicate that chest-worn HR sensors and smartwatches were generally comfortable for preadolescent children.

### Preference for Smartwatch

An interesting finding of this study on the feasibility of smart devices is the appeal of a smartwatch over a smartphone. Researchers did not anticipate all participants to choose a smartwatch over a smartphone for observing their HR information during the study. This is an issue that was not discussed in previous literature [37,38]. Upon reflection, this preference aligns with current commercial trends. Most preadolescent children (9/11, 82%) who participated in this study did not have previous experience with smartwatches or wristwatches in general, which may have contributed to a novelty effect. However, during the interview, they identified the convenience of using a wrist-worn device to view their HR in real time. Several participants (6/11, 54%) commented on perceived ease of use, where they would only have to move their wrists up to be able to view their HR rather than pulling a phone out of a pocket. Other participants (5/11, 45%) favored the security of a worn device, noting that they do not have to worry about dropping a watch compared to a phone. Furthermore, some participants (2/11, 18%) noted that watches may be more comfortable during PA, as a phone in their pocket would move undesirably.

### Usability

Research on the design of mobile apps and the use of commercial wearable sensors has focused on the design process; technical limitations, such as loading speed; and efforts to ensure hardware capabilities and accuracy [39,40]. We developed the Connexx app following best practices based on industry standards and previous research [40,41]. Participants rated the Connexx app with a SUS score of 77, which is above the threshold score of 68 [28], indicating that the app has above-average usability. In addition, the Connexx app received a higher score than other digital health apps, more in line with other PA apps [42]. The usability of the Connexx app was demonstrated by participants throughout the study.

For most participants (9/11, 82%), this was their first time using a smartwatch, and for all participants, this was their first time observing their real-time HR. We helped participants with the initial setup, such as inputting their age and other information in the Connexx app. However, many participants (7/11, 64%) quickly figured out how to operate the Connexx app themselves, and they could input text, navigate the menus, and begin and end workouts on their own without our intervention. This ability not only demonstrated the Connexx app’s usability but also this population’s familiarity with modern technology and its use, which was a concern for developing health-related apps for preadolescent children [43].

Despite the high SUS score, there were issues with the Connexx app. The Connexx app was initially developed for smartphones and then adapted to smartwatches, creating unanticipated usability challenges. The main PA screen for Connexx featured live tracking of several data fields, including HR, activity time, and speed. While this was easy to read on a smartphone display, on a smaller smartwatch display, the presence of multiple data fields resulted in each field being too small to be easily legible during PA. Thus, participants used multiple strategies to stabilize the smartwatch to be able to read what their HR was. The inclusion of a large color-coded portion at the bottom of the smartwatch display was helpful in informing participants of their HR zone at a glance. Despite difficulty reading portions of the app display, participants found it easy and fun to use. Furthermore, some participants (2/11, 18%) indicated that they not only wanted to use the app again in the future but also would like to share it with their friends.

### Other HR Findings

Additional findings from this study indicated that an introductory lesson on HR helped provide participants with a basic understanding of their HR and demonstrated the feasibility of using HR during MVPA. Some participants (2/11, 18%) viewed high HR as an achievement, and the preferences for HR zones are linked to HR achievements for some participants.

Many participants (7/11, 64%) did not have previous experience with HR. An introductory lesson on HR and HR zones was provided for participants at the beginning of the study session, using a simple description of HR zones, including color-coded visual aids (Figure 2) and multiple descriptors that demonstrated how the participants’ bodies might respond to various HR zones (Table 1). Participants subsequently demonstrated an understanding of their HR information while engaged in PA, responding to our queries about their HR with reports of both their HR and the corresponding HR zones. Furthermore, participants recalled the introductory lesson on HR well enough after engaging in PA to describe HR zones during the interview; some repeated the descriptors from the lesson, while others applied the descriptors to their own experiences. These findings suggest that participants were able to quickly grasp the HR concepts taught during the session and that using HR to target MVPA with preadolescent children is feasible.

This understanding of HR is even demonstrated by participants who were unable to keep their HR in zone 2 during activity 2. Participants who did not keep their HR in zone 2 demonstrated their understanding of HR by fully exerting themselves in an attempt to achieve a high HR reading. These participants were more interested in hitting the highest HR and HR zone possible; they viewed higher HR and HR zone as an accomplishment. While participants used the technology in a way not originally intended by the study’s design, it still showed their understanding of HR and its application during PA.

Finally, many participants (8/11, 73%) preferred having a more granular HR zone division, favoring the 5-zone model over the 3-zone option. Some participants (2/11, 18%) equated zone 5 with a higher sense of accomplishment, mirroring the drive to achieve the highest HR possible. The drive for higher values is reflected in commercially available products for PA tracking. Platforms such as Strava [44] reward users by keeping track of their personal records. Furthermore, when a user’s best performance is faster than any other user for a particular segment, the fastest user is rewarded with a crown icon and the title “King of the Mountain” or “Queen of the Mountain.” The desire for high achievement of these participants is likely a reason why gamification and leaderboards are more effective in PA promotion than health benefits [45].

### Limitations

Despite these findings, this study is not without its limitations. First, the participants had never encountered an app such as Connexx before; therefore, the novelty factor of a new measurement offered by the Connexx app may have offset potential shortcomings arising from font size or other use limitations [46]. As participants continue to use the Connexx app, the SUS score may decrease, as the novelty of displaying real-time HR information wears off. However, as participants indicated having little previous experience or education with HR, this highlights an educational opportunity missing from school curricula, as the participants were interested in learning about their HR.

In addition, the small sample size of this feasibility study limits the generalizability of the findings. While there were almost equal numbers of male and female participants, there was little racial or ethnic diversity (9/11, 82% non-Hispanic White). However, the sample size was still sufficient to demonstrate the usability of these HR monitoring technologies with preadolescent children [25]. Future research is needed to explore the technologies with a larger, more diverse sample.

Because this was a feasibility study to explore whether adolescent children could understand and use their HR information in real time as opposed to measuring HR change in response to PA, we did not control for other confounding variables (eg, the child’s fitness level, diet, and resting HR). Rather, as a first step, this study simply examined whether HR use was possible. Moreover, due to the study design, this study did not take advantage of the full scope of capabilities developed as part of the Connexx app, including displaying a paired partner’s HR information in real time. Future studies will further explore these capabilities of the Connexx app.

### Conclusions

This study aimed to investigate the feasibility and usability of multiple technologies to measure and display HR information for preadolescent children. It examined whether preadolescent children could effectively use these devices to view their HR and make practical use of the information. The results indicate that preadolescent children understood and used their HR to regulate their MVPA and enjoyed using Connexx for real-time HR observation. Participant responses demonstrate the ability to use and the desire for access to real-time HR information during PA. Future research should build on these findings regarding the use of real-time HR as an intervention tool to target specific MVPA goals. This could involve testing strategies that use HR and real-time monitoring as direct targets in program designs. Preadolescent children’s interest in using real-time HR should be leveraged by researchers, health professionals, and educators to promote MVPA in an engaging way for this population.

## Appendix

Multimedia Appendix 1 Explanation of heart rate zones.

Multimedia Appendix 2 Semistructured interview script.

## Abbreviations

BPM: beats per minute
HR: heart rate
MVPA: moderate- to vigorous- physical activity
PA: physical activity
REDCap: Research Electronic Data Capture
SUS: System Usability Scale
