# Supplementary material for: Preadolescent Children Using Real-Time Heart Rate During Moderate to Vigorous Physical Activity: A Feasibility Study
Source: JMIR Hum Factors. 2025 Mar 6;12:e58715. doi: 10.2196/58715 (PMC11926448; doi:10.2196/58715)
Supplement: Multimedia Appendix 1 [file humanfactors_v12i1e58715_app1.docx]

HR zones explanation

5 zones for heart rate

Zone 1: 0-120 BLUE 0-60% of max HR 😀

Zone 1 is a very easy, low-intensity heart rate zone. Think sitting on the couch, walking around your home. You can easily talk to other people without thinking about your breathing.

Zone 2: 121-140 GREEN 61-70% of max HR 😐

Zone 2 is a low-intensity effort heart rate zone. Think of this as a very brisk walk, a bike ride, a light jog, or maybe even a steady hike. You can still talk to other people, but you notice that your breathing and have to talk around your breathing a little bit.

Zone 3: 141-160 YELLOW 71-80% of max HR 😬

Zone 3 is a steady, medium-intensity effort heart rate zone. Think of this as a steady jog, a strenuous hike, or a fast-paced bike ride. You are still able to talk to other people, but you can only do so in short sentences, and then you have to catch your breath after.

Zone 4: 161-180 ORANGE 81-90% of max HR 😫

Zone 4 is a high-intensity effort heart rate zone. This will be a very fast run, jumping rope, or other very effortful activities. You are barely able to talk, usually just one or two words at a time. You are breathing very hard so must fit your words in between breaths.

Zone 5: 181-200 RED 91-100% of max HR 😵

Zone 5 is the max-intensity effort heart rate zone. This isn’t something you can be in for any length of time. Your heart rate is usually only in zone 5 for a few seconds at a time as the result of a hard effort while already doing something else active. For instance, if you’re already playing basketball and get into a fast break, or if you’re running and must speed up to catch up to someone. You are unable to talk at zone 5 heart rate as you’ll need to breath really hard and fast.
