# Supplementary material for: Preadolescent Children Using Real-Time Heart Rate During Moderate to Vigorous Physical Activity: A Feasibility Study
Source: JMIR Hum Factors. 2025 Mar 6;12:e58715. doi: 10.2196/58715 (PMC11926448; doi:10.2196/58715)
Supplement: Multimedia Appendix 2 [file humanfactors_v12i1e58715_app2.docx]

Post-Exercise Interview Script

“Great job! Now, we are going to talk a little about your experiences. There are no right or wrong answers, and everything you share with us is very helpful as we figure out what kinds of monitors and tools work best for lots of kids.

1. At the beginning, you picked between a smartphone and smartwatch to watch your heart rate. Why did you pick the one you picked?
2. [ask only if they used a smartwatch] How did the smartwatch feel when you first put it on?
   1. Did the smartwatch feel comfortable or did it bother you?
3. How did the chest strap feel when you first put it on?
   1. Did you notice the chest strap during the activity?
4. What happened during the activity that made you check your heart rate?
   1. Did you like knowing what your heart rate was?
   2. Did you ever feel confused when you saw what your heart rate was?
5. When you were trying to keep your heart rate in zone 2, what did you have to do to keep it in zone 2?
6. Can you explain, in your own words, how your heart rate responds to different levels of activity?
7. Now that you have done the activity, what do heart rate zones mean to you?
8. Today we used 5 heart rate zones during the activity. Would you have liked it better if we had used only 3 zones instead of 5?
   1. Would you have liked using 3 zones instead of 5? Why or why not?
   2. Would you have liked having fewer big zones (3 zones)?
9. Before today’s session, had you ever learned about your heart rate?
   1. What did you learn?
10. Before this study, had you ever used other devices to view your own heart rate? [if so, what devices]?
11. What did you find to be confusing or frustrating about using the app during physical activity?
12. Would you like to use this app again in the future?
    1. How do you think you might use it?
    2. During what kind of activity?
13. Thank you so much for these helpful answers! I have a few more questions for you and then we will be all done. For these next questions, you will be answering them on the screen by sliding the answers from 1 to 5, depending on how much you agree with each one. Here they are: (Children’s SUS)
